# Mapping the Green-Lipped Mussel (*Perna canaliculus*) Microbiome: A Multi-Tissue Analysis of Bacterial and Fungal Diversity

**DOI:** 10.1007/s00284-021-02758-5

**Published:** 2022-01-29

**Authors:** Siming Li, Tim Young, Stephen Archer, Kevin Lee, Shaneel Sharma, Andrea C. Alfaro

**Affiliations:** 1 Aquaculture Biotechnology Research Group, Faculty of Health and Environmental Sciences, School of Science, Auckland University of Technology, Private Bag, 92006, Auckland, 1142 New Zealand; 2 The Centre for Biomedical and Chemical Sciences, Faculty of Health and Environmental Sciences, School of Science, Auckland University of Technology, Private Bag, 92006, Auckland, 1142 New Zealand; 3 Faculty of Health and Environmental Sciences, Auckland University of Technology, Private Bag, 92006, Auckland, 1142 New Zealand

## Abstract

Poor health and mortality events of the commercially important and endemic New Zealand green-lipped mussel (*Perna canaliculus*) pose a threat to its industry. Despite the known importance of microbiomes to animal health and environmental resilience, the host-associated microbiome is unexplored in this species. We conducted the first baseline characterization of bacteria and fungi within key host tissues (gills, haemolymph, digestive gland, and stomach) using high-throughput amplicon sequencing of 16S rRNA gene and ITS1 region for bacteria and fungi, respectively. Tissue types displayed distinctive bacterial profiles, consistent among individuals, that were dominated by phyla which reflect (1) a fluid exchange between the circulatory system (gills and haemolymph) and surrounding aqueous environment and (2) a highly diverse digestive system (digestive gland and stomach) microbiota. *Gammaproteobacteria* and *Campylobacterota* were mostly identified in the gill tissue and haemolymph, and were also found in high abundance in seawater. Digestive gland and stomach tissues were dominated by common gut bacterial phyla, such as *Firmicutes*, *Cyanobacteria*, *Proteobacteria*, and *Bacteroidota*, which reflects the selectivity of the digestive system and food-based influences. Other major notable taxa included the family *Spirochaetaceae*, and genera *Endozoicomonas*, *Psychrilyobacter*, *Moritella* and *Poseidonibacter*, which were highly variable among tissue types and samples. More than 50% of fungal amplicon sequence variants (ASVs) were unclassified beyond the phylum level, which reflects the lack of studies with marine fungi. However, the majority of those identified were assigned to the phylum *Ascomycota*. The findings from this work provide the first insight into healthy tissue microbiomes of *P. canaliculus* and is of central importance to understanding the effect of environmental changes on farmed mussels at the microbial level.

**Supplementary Information:**

The online version contains supplementary material available at 10.1007/s00284-021-02758-5.

## Introduction

The New Zealand green-lipped mussel (*Perna canaliculus*) is an endemic bivalve commonly found within intertidal and subtidal coastal habitats. Mussel beds provide important ecological functions, such as removing suspended sediment and particulate organic material, resulting in improved water quality. *P. canaliculus* is also a highly valued species for the New Zealand’s growing aquaculture industry, which supports a mussel sector worth over NZ$300 million in export revenues [1].

Given their ecological and economic importance, monitoring health of wild mussels and maintaining the health of domesticated stocks is of utmost importance. While infections from pathogenic microbes may lead to deleterious outcomes, host-microbe interactions are also thought to play a key role in maintaining mussel health and organ-level functioning. Filter-feeding marine mussels are in continuous and direct contact with a dynamically shifting microbial environment [2, 3]. Indeed, the high filter feeding capability of marine mussels allows them to filter large volumes of seawater, while capturing different types of particulate water-borne pollutants as well as microorganisms [4, 5]. Growing evidence suggests that microbes can offer their host organisms probiotic functions, such as enhanced pathogen defence, immunological regulation, and improved digestion efficiency and nutrient uptake, among other factors [6–8]. Thus, characterizing the microbial structures of host compartments can inform the underlying functionalities of host-microbiota interactions and associations.

Although a previous report of microbiota characterizations of marine mussels (*Mytilus galloprovincialis*) revealed that different tissues harbour unique microbial communities which serve specific functional purposes [9], the functions of microbial communities in the gut, stomach, and digestive gland tissues are less explored among the diverse range of marine molluscs. Additionally, there have been no microbiota characterization studies for the endemic New Zealand green-lipped mussel *P. canaliculus*. The aim of this study was to profile the microbiota associated with different tissues of farmed green-lipped mussels using high-throughput sequencing. The main objectives were to: (1) profile marine bacteria and fungi in different mussel tissues and the surrounding seawater, (2) describe microbiome variability among individual samples, (3) determine bacterial and fungal community similarity/dissimilarity among the different tissue types, and 4) Identify key dominant host-associated taxa across the tissue types.

## Material and Methods

### Sample Collection

Five healthy adult mussels (length = 95.8 mm ± 6.6; weight = 66.3 g ± 9.9) and a sample of seawater (1 L) were collected in September 2020 (autumn) from a mussel farm located in Kaiaua, Firth of Thames, New Zealand (GPS coordinate: − 37.0610, 175.3002). Mussels were cleaned and washed externally with fresh filtered seawater to remove biofouling. Haemolymph was extracted from the abductor muscle using sterile disposable 1 mL syringes and transferred to sterile 2 mL cryovials (BioStor™) containing 20 µL RNA stabiliser (Qiagen, Germany). The digestive gland, stomach and gill tissues were dissected and the samples were placed in 2 mL cryovials with RNA stabiliser (200 µL), then immediately snap-frozen in liquid nitrogen and stored at − 80 °C until further analyses. Sub-aliquots of seawater were filtered through single use 25 mm diameter Whatman filters with 0.2 µm pore size (Cytiva, USA) using 20 mL syringes flooded with RNA stabiliser. Filters were sealed in parafilm and stored at 4 °C for 2 weeks before DNA extractions.

### Microbial DNA Extraction

Total microbial DNA was extracted from tissue samples (each 20–30 mg) and haemolymph (200 μL) using the DNeasy PowerSoil kit (Qiagen, Germany) according to the manufacturer's instructions and the adapted protocol of Musella et al. (2020). Tissues were lysed using a FastPrep system (MP Biomedicals; Irvine, California) at six movements per second for one minute prior to extraction. The elution step from the DNeasy PowerSoil kit was repeated twice with 50 μL Tris elution buffer, incubating the columns for five minutes at room temperature before centrifugation. DNA samples were stored at − 20 °C before subsequent processing. To extract microbial DNA from seawater filters, samples were flooded with 1 mL of extraction buffer 1, incubated at 60 °C for 30 min. Then, the fluid was pushed into a clean 2 mL bead tube for processing with the DNeasy PowerSoil kit according to the manufacturer’s instructions. Multiple tubes of seawater were pooled at the column stage.

### PCR Amplicon and Sequencing

Purified DNA samples were quantified using a Qubit 2.0 Fluorometer (Invitrogen; USA). MiSeq (Illumina, USA) libraries were prepared as per manufacturer’s protocol (16S Metagenomic Sequencing Library Preparation; Part # 15044223; Rev. B [Illumina; San Diego, CA, USA]) and as previously described (Archer et al. 2020). PCR was conducted with primer sets targeting the V3-V4 regions of the bacterial 16S rRNA gene: PCR1 forward (5′ CCTACGGGNGGCWGCAG 3′) and PCR1 reverse (5′ GACTACHVGGGTATCTAATCC 3′) and the internal transcribed spacer region (ITS) between the fungal 18S and 5.8S rRNA genes: ITS1 forward (5′-CTTGGTCATTTAGAGGAAGTAA-3′) and ITS2 reverse (5′ GCTGCGTTCTTCATCGATGC 3′).

### Bioinformatics and Statistical Analysis

Data were pre-processed using our established workflow [10]. Briefly, 16S rRNA gene and fungal ITS1 amplicons were processed using the R package DADA2 v1.8 [11] and cutadapt v3.4 [12] to remove forward (CCTACGGGNGGCWGCAG) and reverse (GACTACHVGGGTATCTAATCC) primer sequences for 16S rRNA gene, and forward (CTTGGTCATTTAGAGGAAGTAA) and reverse (CTTGGTCATTTAGAGGAAGTAA) primer sequences for fungal ITS1 region. High quality bacterial reads (forward base reads < 230 and reverse base reads < 220 were trimmed and removed) were then clustered into amplicon sequence variants (ASVs) which were assigned taxonomic ranks using SILVA nr v132 database [13]. Fungal reads were taxonomically classified using the UNITE v7.2 database [14]. R v3.5.2 [15] and the R packages MicrobiomeAnalyst [16], phyloseq [17], and ggplot2 [18] were used for downstream statistical analysis and data visualisation (i.e., relative bacterial/fungal abundances, ordination [principal coordinates analysis; Bray–Curtis], hierarchical cluster analysis [Bray–Curtis distance; Ward linkage]), alpha diversity [Chao1 index], heatmap [data scaled by pseudo log]).

## Results & Discussion

**Fig. 1:**
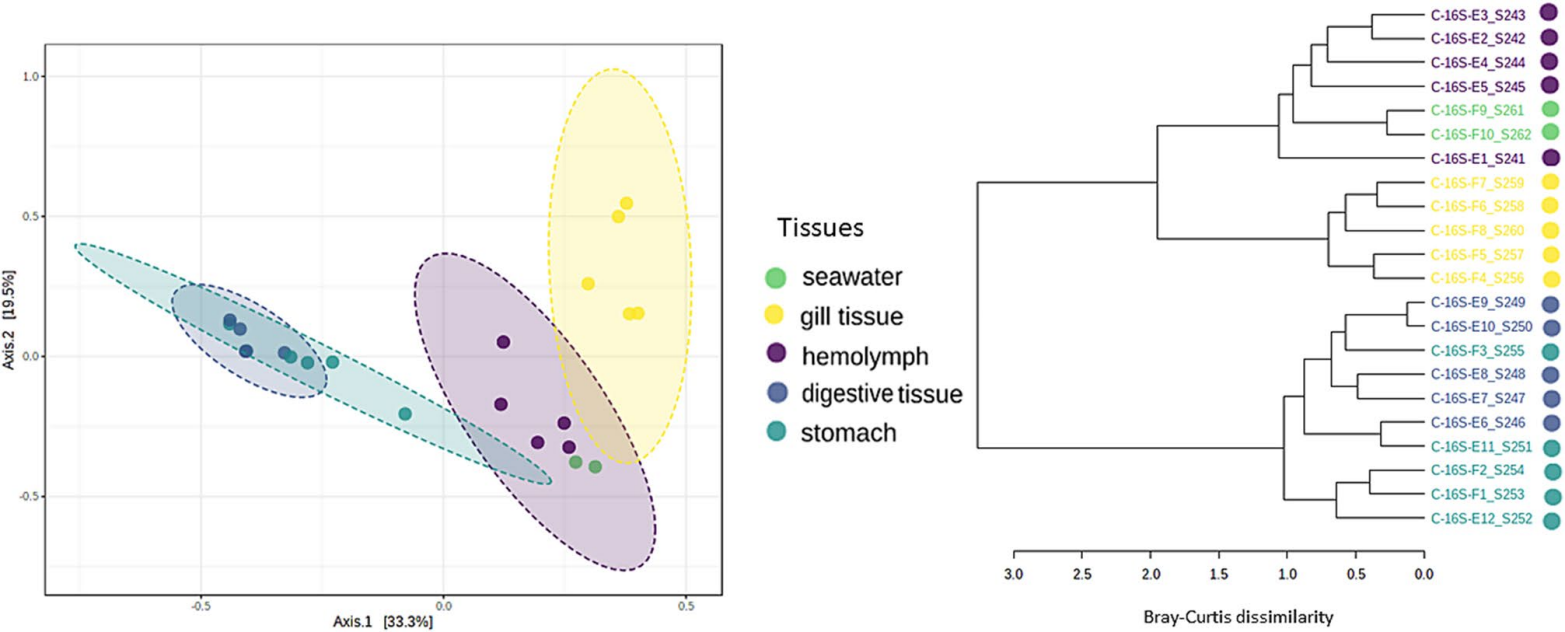
Cluster analyses of *P. canaliculus* tissues and seawater based on bacterial profiles (ASV) and Bray–Curtis distances: **a** Principal coordinates analysis (PCoA); **b** Hierarchical clustering dendrogram (Ward algorithm). Both constructed based on Bray–Curtis distances

**Fig. 2:**
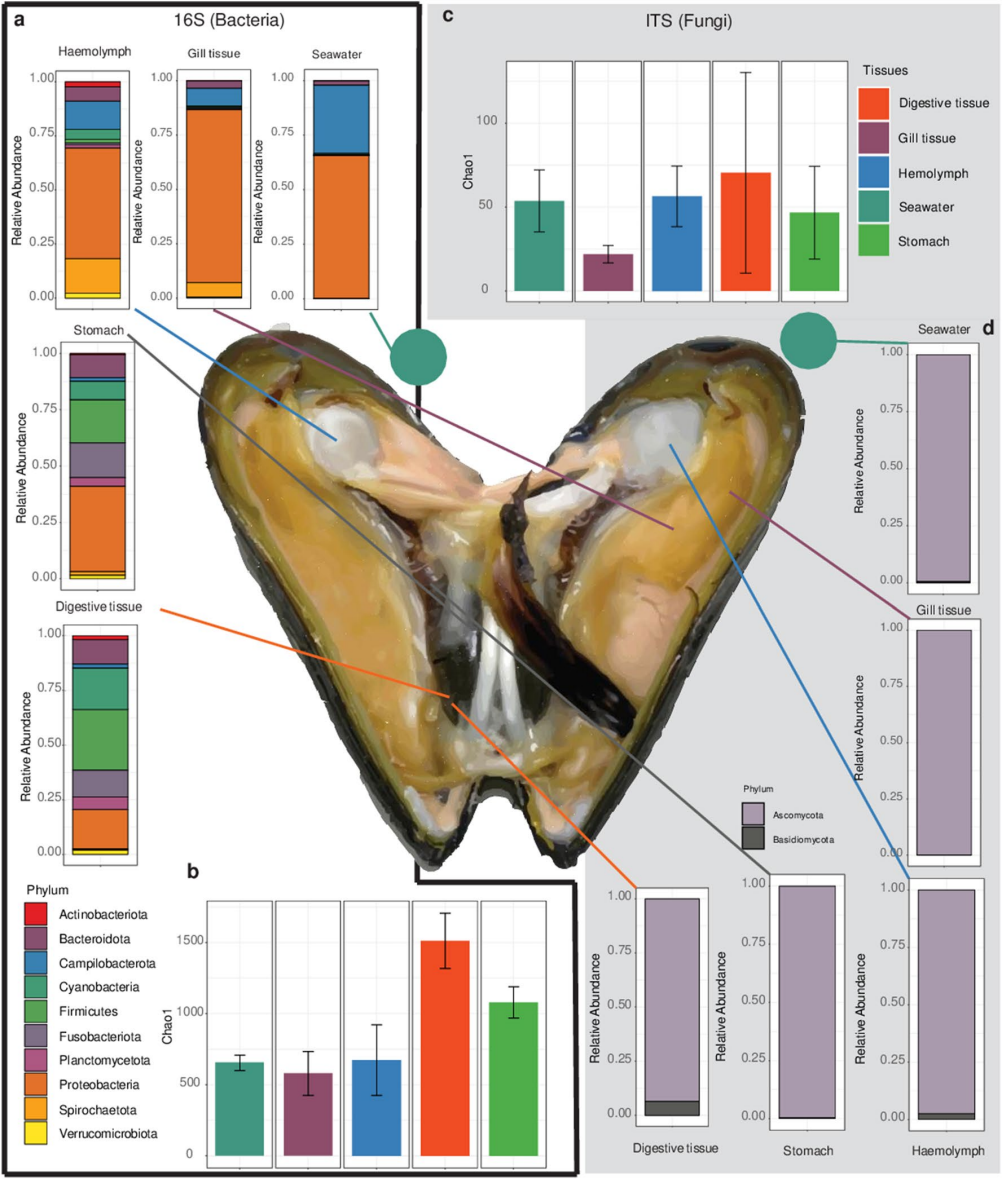
Microbiome profiling of *P. canaliculus*: **a** Bacterial relative abundances at the phylum level in different tissue types and seawater; **b** Bacterial alpha diversity; **c** Fungal alpha diversity; **d** Fungal relative abundances at the phylum level in different tissue types and seawater

Bacterial communities were distinct by tissue type (PERMANOVA; *F* value = 6.1784; *R*^2^ = 0.59246; *P* value < 0.001) (Fig. 1a, b) with the exception of stomach and digestive gland tissues that were highly similar to one another, but distinct from the gill tissues and haemolymph. The seawater samples were also clustered closely with the gill tissue and haemolymph, indicating similar bacterial communities. The gills of bivalves perform respiratory, excretory, and feeding functions, which require them to interact directly with seawater. Therefore, our results suggest that close contact between gills and haemolymph allows waterborne microbiota from the external environment to be transferred to the haemolymph via the gills as has been shown previously [19]. Higher species richness were observed in digestive gland and stomach tissues compared to seawater, haemolymph and gill tissues (*P* value: 0.017667; [ANOVA] *F* value: 4.034) (Fig. 2b). This is most likely reflective of the difference in functions and selection of a host-associated microbiota of symbionts with nutrition-related roles [20, 21]. These findings indicate a highly selective host recruitment of the mussel microbiome which aligns with previous studies in fish; gill microbial communities tend to be more associated with interactions and communication processes involving the circulatory system, signal transduction, and cell motility, whereas gut microbiota are associated with metabolism and genetic information processing [22].

Profiling the microbiome of *P. canaliculus* at multiple taxonomic levels revealed distinct bacterial community structures (Fig. 2a) (for family, class, order, genus levels refer to Supplementary Fig. S1–S5; for significance values of the phyla refer to Supplementay Table 1). The dominance of *Proteobacteria* (*P* value = 1 × 10^–3^; *q* value = 2 × 10^–3^), specifically gamma, and *Campylobacterota* (*P* value = 1.12 × 10^–5^; *q* value = 7.06 × 10^–5^) (a new phylum that contains *Epsilonproteobacteria* based on Genome Taxonomy Database) in the gill tissues and haemolymph are consistent with prior findings in other mussel species [23, 24], oysters [25], and abalone [26]. These findings suggest that host-associated bacterial community in distantly related marine molluscs may be more tightly linked with general tissue types, as a potential consequence of organ-level function and/or environmental interaction. *Proteobacteria* have been found to dominate fish gill tissues where they are thought to play crucial roles in supporting the mucosa’s microbial barrier, and, with many being opportunistic pathogens, they may even contribute to the development and maintenance of the host immune system through stimulatory mechanisms [27, 28]. However, their functional roles, if any, are yet to be established in molluscan gill mucosa.

In regard to the bacterial profile of digestive gland and stomach tissues, the high abundances of anaerobic phyla *Firmicutes* (*P* value = 1 × 10^–3^; *q* value = 6 × 10^–4^) and *Bacteroidota* (*P* value = 0.02; *q* value = 0.03), as well as *Cyanobacteria* (*P* value = 3 × 10^–4^; *q* value = 1 × 10^–3^) were to be expected. Microbes within the phylum *Firmicutes* can produce short-chain fatty acids from complex polysaccharides, which provide nutrition for the intestinal mucosal cells [29, 30]. High levels of *Firmicutes* may also contribute to the maintenance of the normal function of the intestinal mucosa and the regulation of the intestinal microbial environment [29, 31]. *Bacteroidetes* participate in carbohydrate transport and protein metabolism, which are involved in digestive processes [32]. Interestingly, *Bacteroidota*/*Firmicutes* ratios have been extensively researched as markers for gut health and dysbiosis in humans and mice [33–36], however, their significance in mussel gut functioning is yet to be explored. The high abundances of *Cyanobacteria* in digestive gland and stomach tissues in the present study are most likely derived from the environment (ingested food and seawater). However, the absence of this group of bacteria in the gills and haemolymph could be due to said tissues’ undesirable conditions for proliferation of this type of bacteria.

In contrast to bacterial communities, the fungal profiles in this study were more ambiguous due to the large amounts of unmatched/unidentified ASVs. Furthermore, the relative abundances of identified fungi did not reveal any specific patterns or tissue-specific associations (Supplementary information S6–S10). Identified fungal phyla were almost entirely dominated by *Ascomycota*, except for seawater, which contained a large proportion of unidentified phyla. The alpha diversity for fungal species revealed a slightly higher diversity in the digestive gland, and lower diversity in the gills compared to stomach, seawater and haemolymph (Fig. 2c). The lack of clear trends in the distribution of fungi found in this study is not surprising given the stochastic nature of fungal dispersion [37, 38] and the lack of studies on marine fungi [39].

**Fig. 3:**
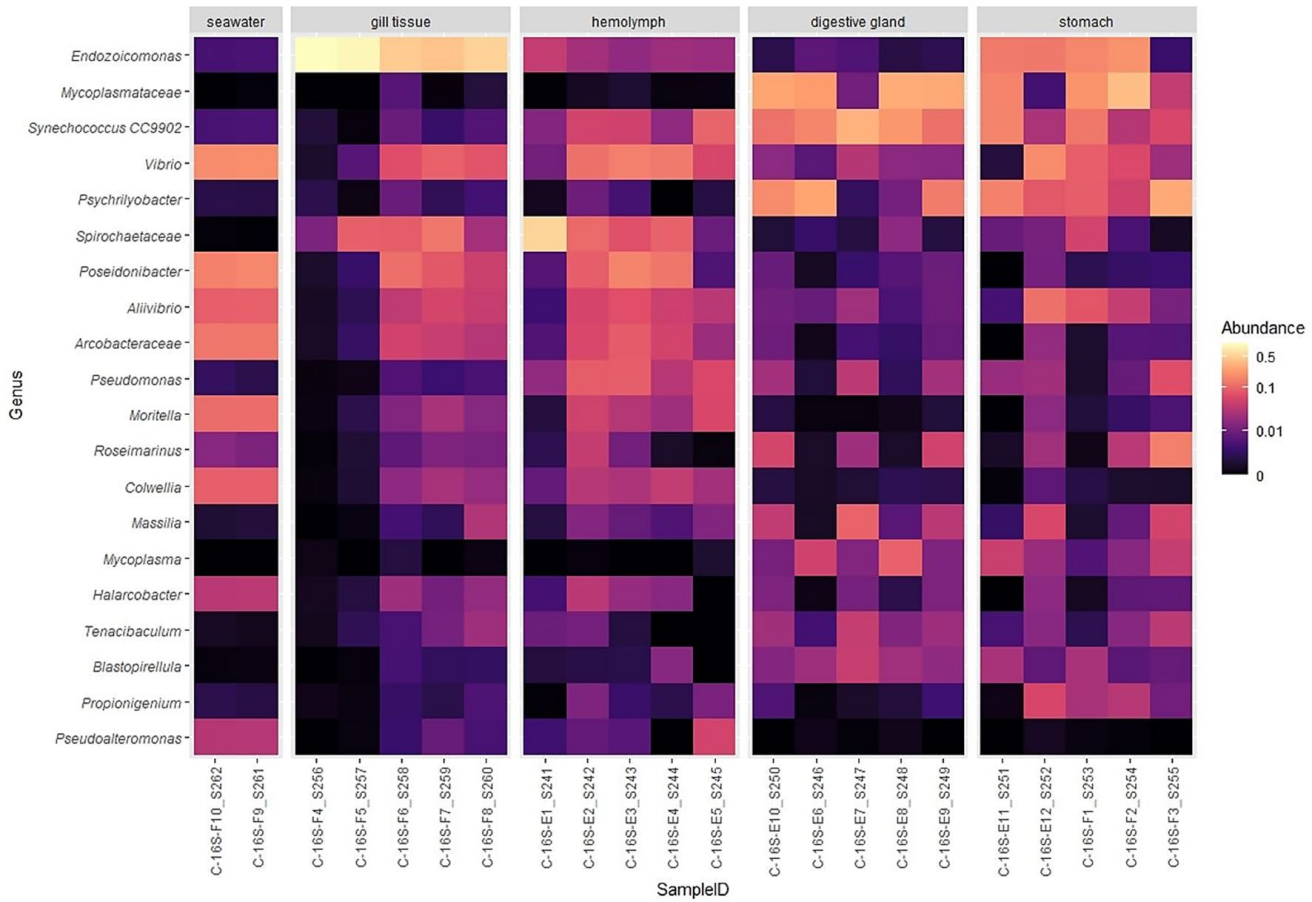
Top 20 relative abundant bacterial genera (ranked from most abundant to least abundant) across different tissue of *P. canaliculus* and seawater. Bacterial genus is shown row-wise, samples are shown column-wise and coloured by relative abundances. The range of the scale has been transformed via a pseudo log transformation. The few family names represent all the genera within that family merged into a single taxon

Finally, to describe microbiome variability among individuals, and to identify key dominant host-associated taxa across tissue types, the top 20 bacterial genera were ranked from highest to lowest in terms of abundances across all samples (Fig. 3). The results visualized via a heatmap revealed that bacterial genera, such as an unclassified genera of families *Spirochaetaceae*, *Moritella* and *Poseidonibacter* were more abundant in gill tissue, haemolymph and/or seawater. Bacteria, such as *Mycoplasma*, *Synechococcus* and *Psychrilyobacter* were elevated in digestive gland and stomach tissues. Interestingly, high relative abundance of *Vibrio* spp. was observed across seawater and all tissue types. The presence of *Vibrio* spp. is to be expected as they are ubiquitous in marine and estuarine environments, and on surfaces and intestinal contents of marine animals [40]. Although many *Vibrio* species are harmless, several can be highly pathogenic for humans and/or marine animals [41–44]. Warm temperature favours the proliferation of *Vibrio* spp. and has contributed to mass mortalities in shellfish farms [45, 46]. Higher abundances of *Moritella* and *Poseidonibacter* in the gill tissues were expected because these bacteria are of marine origin [47–49].

Higher abundances of *Mycoplasma* in the stomach were not surprising as they are common members of the intestinal bacterial flora of many marine species (e.g., fish, abalone) where they may provide nutrients to their hosts [50–52]. *Cyanobacterium Synechococcus* are one of the most important components of photosynthetic picoplankton [53–55], and their presence in digestive samples of *P. canaliculus* represents their dietary origin*. Psychrilyobacter* is a marine member of the phylum *Fusobacteria*. This genus is an obligate anaerobic halophile that is able to grow well in low temperatures, and it has been recently isolated and described from marine sediments and marine animals [56–58]. Interestingly, the most abundant genus identified (*Endozoicomonas*) across samples was elevated in gill and stomach tissues. A study using comparative analysis revealed that *Endozoicomonas* species are likely to participate in nutritional symbiosis and their genomes may be enriched for transport and secretion processes, such as transfer of carbohydrates, amino acids, and proteins between the symbiont and host [59]. In addition, *Endozoicomonas* species seem to have symbiotic relationships with the host by producing antimicrobial substances to deter potential invading microbes [60]. Previous reports have also shown that *Endozoicomonas* dominates the gut of *M. galloprovincialis* in response to thermal stress (27 °C), suggesting that the microbes from this genus play a crucial role in maintaining health [61]. Contrary to these reports, the presence of *Endozoicomonas* has been associated with mortalities of shellfishes, such as green-lipped mussels, clams and scallops in New Zealand [62], and infecting the gill tissues of king scallop [63]. The identification of major microbial genera in *P. canaliculus* microbiomes demonstrates key associations and similarities with other marine organisms. These taxa also represent targets for future microbial-host interaction research in *P. canaliculus* for the potential development of host health biomarkers.

## Conclusions

Marine bacteria and fungi were profiled in different tissues of *P. canaliculus* and surrounding seawater. Distinct compositional patterns of microbes were identified at various taxonomic levels. Seawater, gills, and haemolymph contained *Proteobacterial* groups, while digestive gland and stomach tissues were dominated by common anaerobic gut microbes involved in fatty acid synthesis, carbohydrate digestion and gut maintenance. Fungal profiles in all samples were dominated by taxa within the phylum *Ascomycota*, but could not be identified beyond this taxonomic level. This study also highlights the open association between the circulatory physiology (gills and haemolymph) of mussels and surrounding seawater, and the high selectivity of microbiomes in the digestive system (digestive gland and gut). Furthermore, by comparing individual sample variability, we identified key genera of interest, such as *Endozoicomonas*, which could potentially be used as markers for mussel health in the future. Our study represents the first detailed characterization of microbiome profiles of *P. canaliculus* within different tissues, hence providing a baseline for future physiological and health studies of this important aquaculture species.

## Supplementary Information

Below is the link to the electronic supplementary material.

Supplementary file1 (DOCX 1805 kb)

## Data Availability

All sequence data are published in NCBI BioProject (Accession: PRJNA788989). Raw data and outputs may also be available upon request.
