# Supplementary material for: Mapping the Green-Lipped Mussel (Perna canaliculus) Microbiome: A Multi-Tissue Analysis of Bacterial and Fungal Diversity
Source: Curr Microbiol. 2022 Jan 29;79(3):76. doi: 10.1007/s00284-021-02758-5 (PMC8799583; doi:10.1007/s00284-021-02758-5)
Supplement: Supplementary file 1 — Supplementary file1 (DOCX 1805 kb) [file 284_2021_2758_MOESM1_ESM.docx]

**Supplementary Information**

**Table 1.** Mann-Whitney/Kruskal-Wallis analysis on the differences in relative abundance of the identified phyla between the tissue groups.

|  | Pvalues | FDR | Statistics |
| --- | --- | --- | --- |
| Desulfobacterota | 3.13E-06 | 3.38E-05 | 19.865 |
| Myxococcota | 3.56E-06 | 3.38E-05 | 19.494 |
| Campilobacterota | 1.12E-05 | 7.06E-05 | 16.437 |
| Acidobacteriota | 1.69E-05 | 8.01E-05 | 15.427 |
| Firmicutes | 0.00017073 | 0.00064877 | 10.594 |
| Cyanobacteria | 0.00035269 | 0.0011168 | 9.3274 |
| Planctomycetota | 0.00072052 | 0.0019144 | 8.1792 |
| Nitrospirota | 0.00080604 | 0.0019144 | 8.0074 |
| Verrucomicrobiota | 0.0009727 | 0.0020535 | 7.7243 |
| Proteobacteria | 0.0013536 | 0.0025718 | 7.2409 |
| Chloroflexi | 0.0028945 | 0.0049995 | 6.1955 |
| Actinobacteriota | 0.0032605 | 0.0051624 | 6.0396 |
| Fusobacteriota | 0.012783 | 0.018683 | 4.3903 |
| Bacteroidota | 0.020349 | 0.027616 | 3.8821 |
| Bdellovibrionota | 0.26651 | 0.3238 | 1.4311 |
| Fibrobacterota | 0.27267 | 0.3238 | 1.4111 |
| Spirochaetota | 0.40605 | 0.45382 | 1.0606 |
| Not_Assigned | 0.51602 | 0.54469 | 0.84474 |
| Patescibacteria | 0.64356 | 0.64356 | 0.63635 |


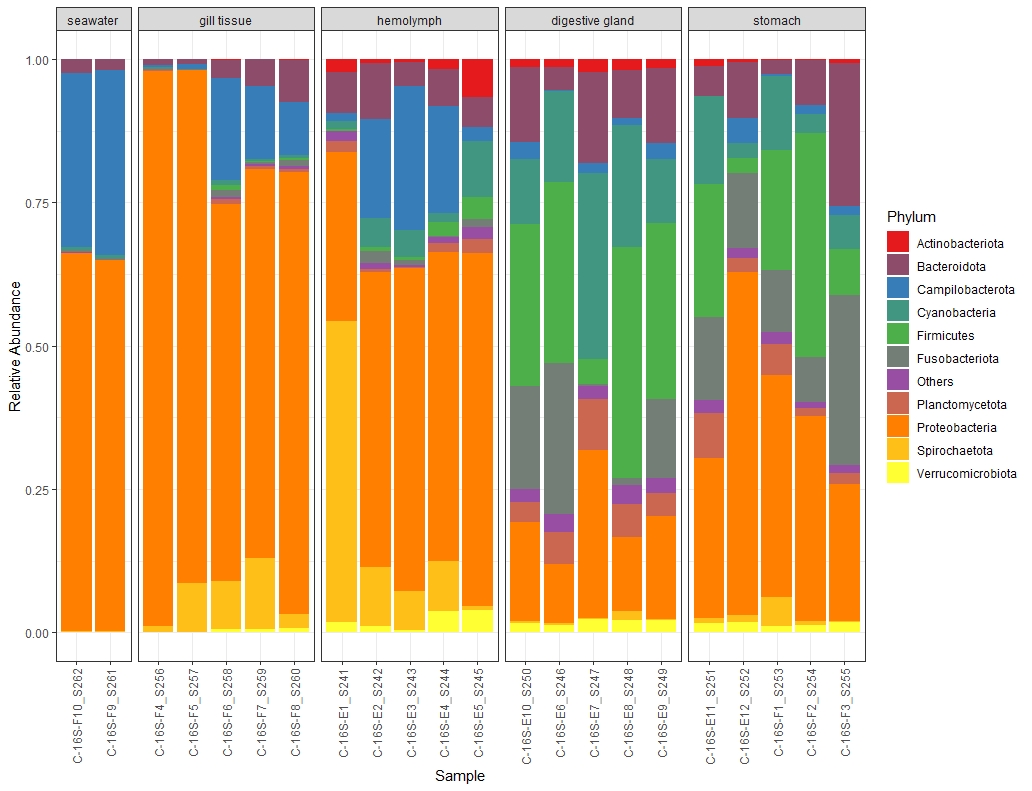


**S1** Stacked barplot of relative abundance of bacteria at the phylum level across all tissue groups.


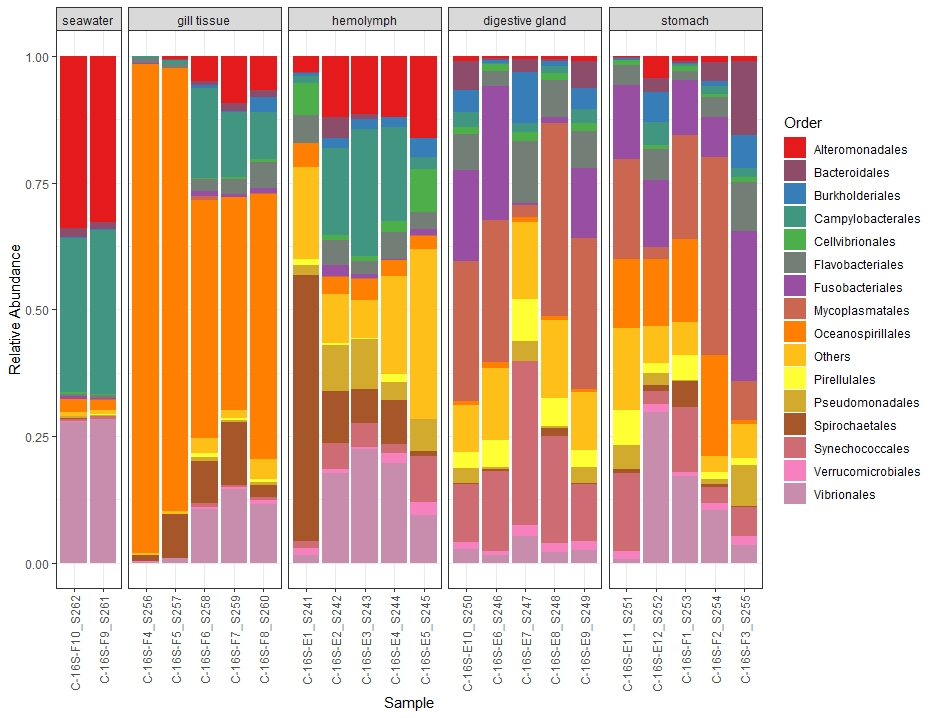


**S2** Stacked barplot of relative abundance of bacteria at the order level across all tissue groups.


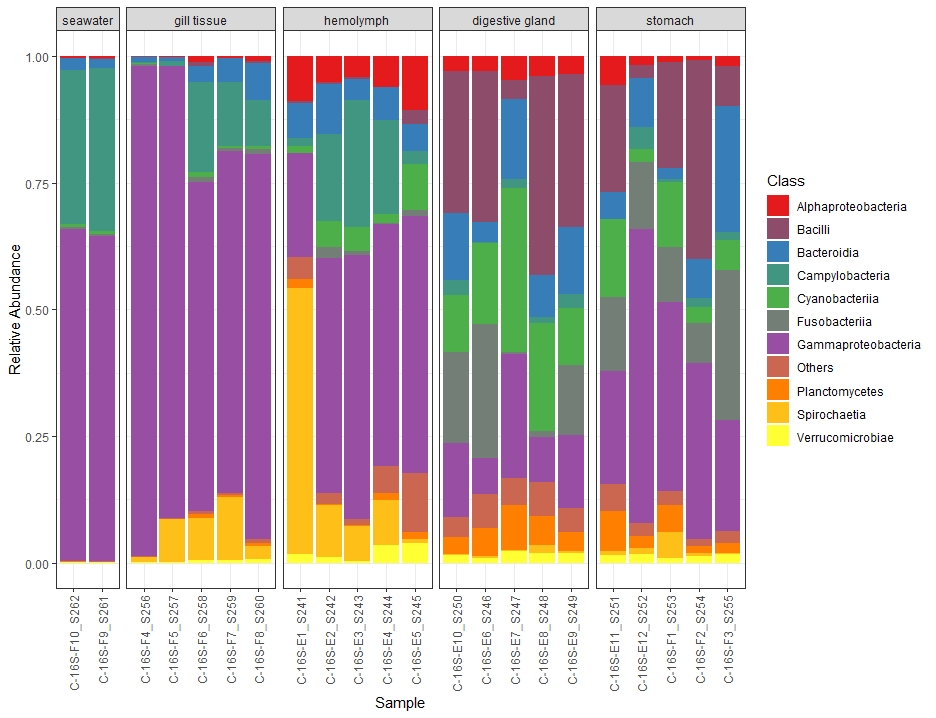
**S3** Stacked barplot of relative abundance of bacteria at the class level across all tissue groups.


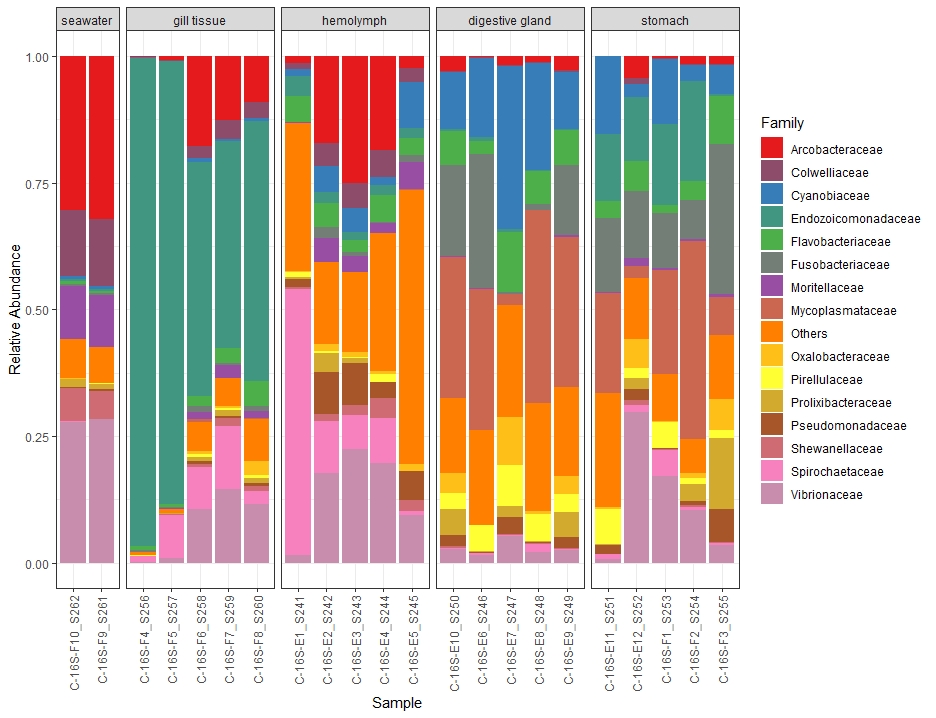


**S4** Stacked barplot of relative abundance of bacteria at the family level across all tissue groups.


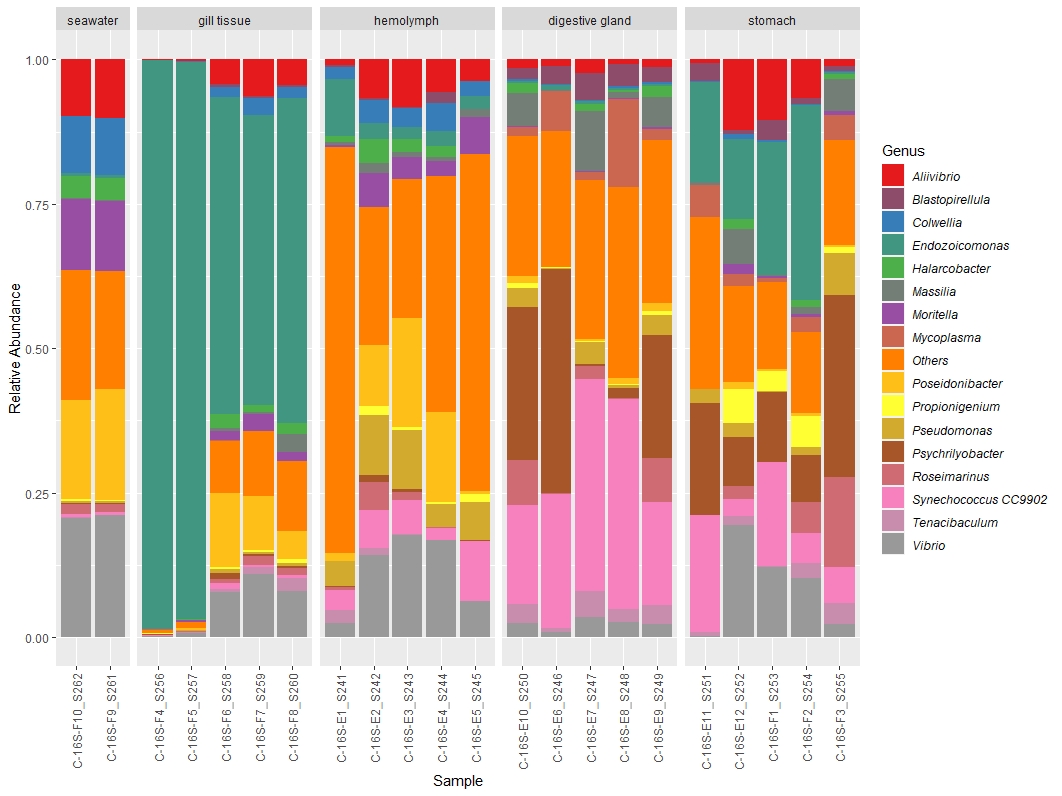


**S5** Stacked barplot of relative abundance of bacteria at the genus level across all tissue groups.


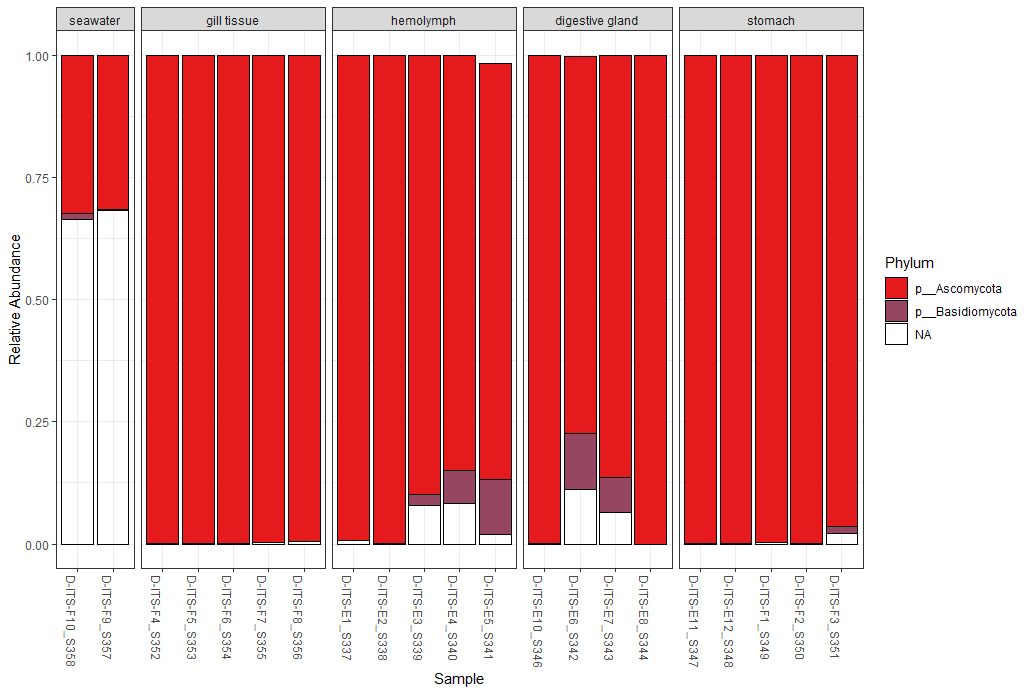


**S6** Stacked barplot of relative abundance of fungus at the Phylum level across all tissue groups. NA represents unclassified fungus ASVs.


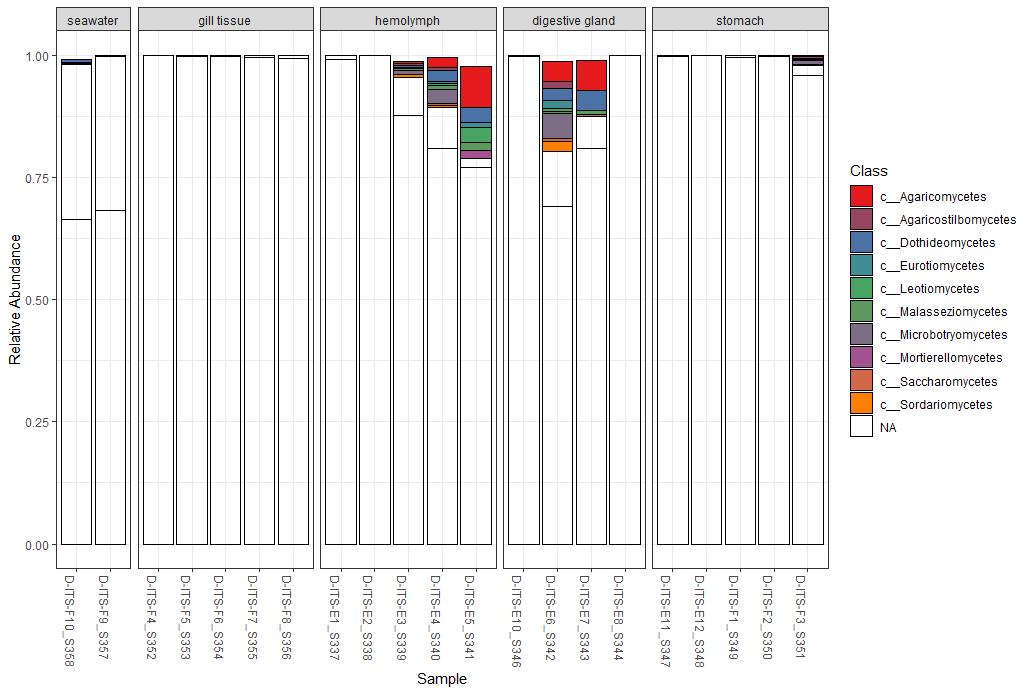


**S7** Stacked barplot of relative abundance of fungus at the class level across all tissue groups. NA represents unclassified fungus ASVs.


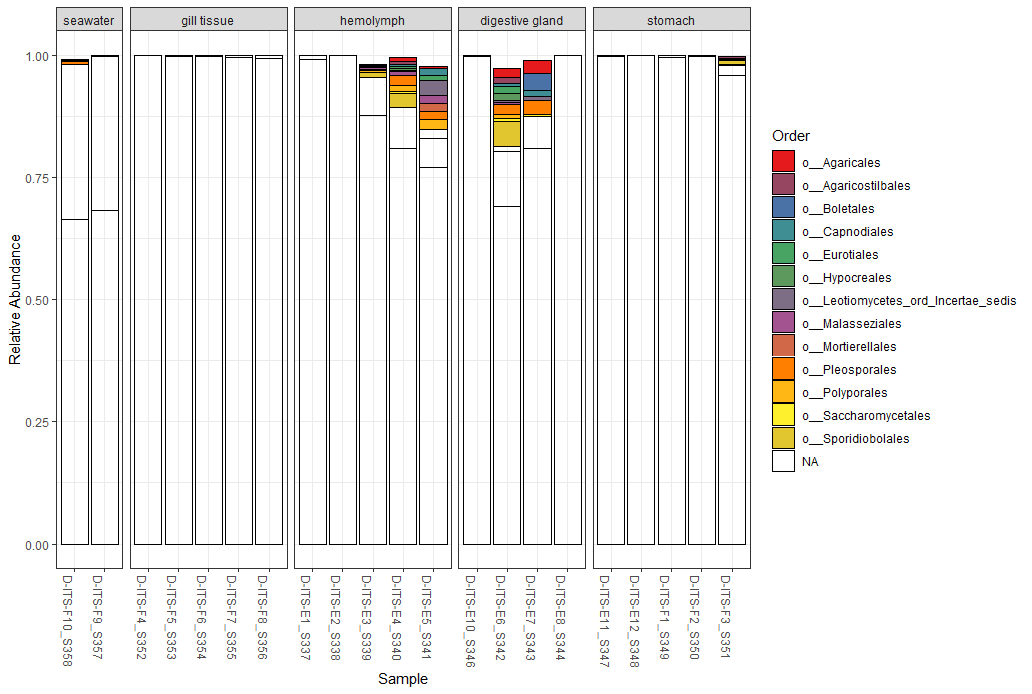


**S8** Stacked barplot of relative abundance of fungus at the order level across all tissue groups. NA represents unclassified fungus ASVs.


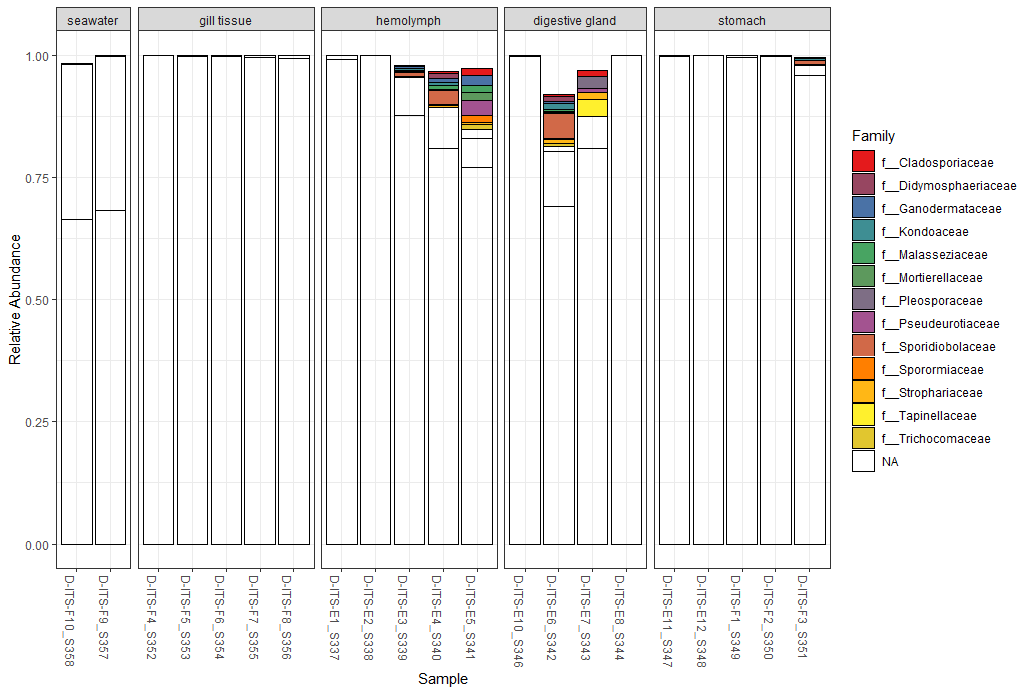


**S9** Stacked barplot of relative abundance of fungus at the family level across all tissue groups. NA represents unclassified fungus ASVs.


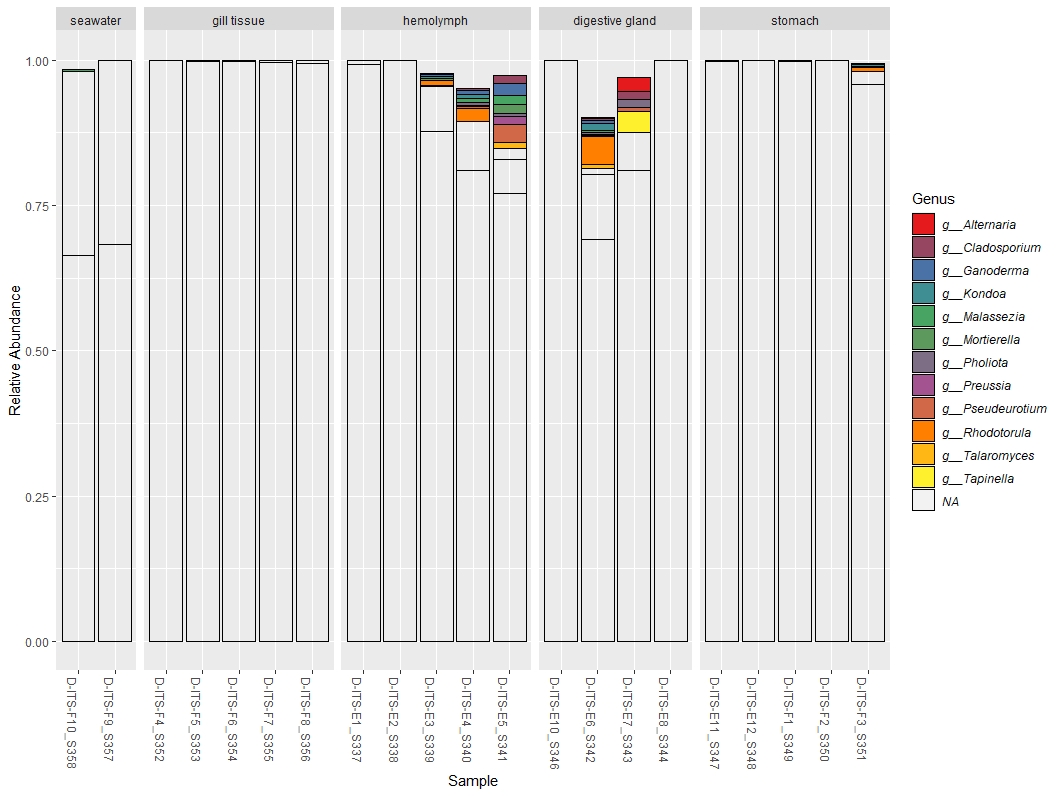


**S10** Stacked barplot of relative abundance of fungus at the genus level across all tissue groups. NA represents unclassified fungus ASVs.
